# Associations between generative AI usage orientations and critical thinking disposition among college students in China

**DOI:** 10.3389/fpsyg.2026.1957029

**Published:** 2026-09-28

**Authors:** Shuai Yin, Yitao Gan, Qing Zhao

**Affiliations:** 1 School of Economics and Management, Weifang Engineering Vocational College, Qingzhou, China; 2 School of Educational Science, Yangzhou University, Yangzhou, China; 3 Office of Science and Technology, Weifang Engineering Vocational College, Qingzhou, China

**Keywords:** college students, critical thinking disposition, GenAI usage orientations, generative artificial intelligence, higher education

## Abstract

**Introduction:**

Research on generative artificial intelligence (GenAI) in higher education commonly describes use in terms of adoption or frequency. Students, however, may engage with the same systems in different ways.

**Methods:**

This study conceptualises three GenAI usage orientations (Tool, Guidance, and Collaboration) and surveys 430 college students in China to examine their bivariate and adjusted associations with critical thinking disposition (CTD). Tool represented comparatively delegative use, including task completion centred on GenAI and direct adoption of its output with limited independent revision. Guidance reflected instrumental requests for explanations, examples, clues, or diagnostic assistance. Collaboration reflected iterative coordination directed by students. Based on the measurement evaluation, CTD was used as the primary outcome.

**Results:**

Tool showed no statistically significant bivariate association with CTD (*r* = −0.044, *p* = 0.363) but had a negative adjusted association (*β* = −0.189, *p* < 0.001). This pattern is consistent with statistical suppression; the adjusted Tool coefficient represents a conditional association. Guidance (*β* = 0.126, *p* = 0.017) and Collaboration (*β* = 0.321, *p* < 0.001) showed positive adjusted associations. The orientation block explained an additional 10.37% of the variance in CTD, and the final model explained 38.95% of CTD variance.

**Discussion:**

The findings show that behavioural orientations provide information beyond general measures of GenAI experience.

## 1 Introduction

Generative artificial intelligence (GenAI) now supports many academic activities in higher education. Students use conversational systems to clarify concepts, generate ideas, revise text, and assist with research (Chan and Hu, 2023; Johnston et al., 2024). Their acceptance varies by purpose. For example, students report greater acceptance of grammar support than of complete essay generation (Johnston et al., 2024). They also value personalised feedback while remaining concerned about accuracy, privacy, and excessive reliance (Chan and Hu, 2023). These distinctions suggest that educational consequences may depend on how students organise their use of GenAI.

This issue is pertinent to critical thinking disposition (CTD), which refers to the inclination to question claims, consider alternatives, draw on evidence, and reflect on judgement (Sosu, 2013). GenAI can supply explanations and alternative perspectives, yet fluent output may also invite premature acceptance. Studies of scientific inquiry and mathematics show that unrestricted support can reduce effort or weaken subsequent unaided performance, whereas structured guidance can limit these costs (Bastani et al., 2025; Stadler et al., 2024). Other research reports a positive association between student interaction with ChatGPT and critical reasoning performance (Suriano et al., 2025). Although these studies assessed task performance rather than CTD, they indicate that cognitive consequences may vary with the organisation of support and interaction.

Survey research commonly records adoption, frequency, attitudes, or perceived value (Chan and Hu, 2023; Johnston et al., 2024). These indicators capture general exposure and evaluation. They cannot identify how students organise task work and evaluative responsibility with GenAI. Reliance measures distinguish reflective, cautious, thoughtless, and collaborative responses to AI output during problem solving (Hou et al., 2025b). Instrumental help-seeking and collaborative AI literacy describe further forms of engagement (Chen and Law, 2025; Sidra and Mason, 2026). Together, these studies identify relevant behaviours. They do not provide a common account of delegative task completion, targeted assistance, and iterative coordination across general academic activities.

Earlier classifications provide conceptual background for this distinction. Taylor (1980) described computers as tutor, tool, or tutee according to their relationship with learners. Mollick and Mollick (2023) later outlined several educational roles for conversational AI, including tutor, coach, teammate, and simulator. These accounts highlight variation in how learners engage with technological support and retain responsibility for academic work. Within this conceptual framing, the present study distinguishes Tool, Guidance, and Collaboration orientations. The constructs were operationalised using items adapted from published measures of GenAI reliance, instrumental help-seeking, and collaborative AI literacy (Chen and Law, 2025; Hou et al., 2025b; Sidra and Mason, 2026). Tool reflects delegative use, in which students rely on GenAI to complete parts of a task or adopt its output with limited independent evaluation or revision. Guidance concerns seeking explanations, examples, clues, or diagnostic assistance while the student retains responsibility for understanding. Collaboration concerns iterative coordination in which the student refines prompts, evaluates responses, and integrates GenAI contributions. The orientations are continuous, nonexclusive, and may coexist across academic activities.

Evidence remains limited on how these three orientations relate to CTD when considered together. Hou et al. (2025a) linked students’ critical thinking characteristics with several forms of GenAI reliance during problem solving. Their model positioned critical thinking before reliance. The present study addresses a complementary question by treating CTD as the statistical outcome. The cross-sectional design does not establish temporal order. Existing CTD may shape students’ orientation patterns. Repeated patterns of GenAI use may also relate to later CTD. The analysis first estimates the bivariate association of each orientation with CTD. It then estimates their mutually adjusted associations after accounting for demographic characteristics, general GenAI experience, and overlap among the orientations. Accordingly, the study explored two research questions:

1) What are the bivariate associations between each GenAI usage orientation and CTD?
2) What are the mutually adjusted associations of the three orientations with CTD, and how much additional variance do they account for beyond demographic characteristics and general GenAI experience?

## 2 Literature review and theoretical framework

### 2.1 Generative AI use and critical thinking disposition

GenAI is increasingly present in higher education, where students use it to locate information, request explanations, generate drafts, test arguments, and refine solutions (Miao and Holmes, 2023). These activities differ in how task work and evaluation are organised between student and system.

CTD refers to a relatively stable willingness to approach information, claims, and problems through questioning, reflection, and attention to evidence (Halpern, 1998; Loyens et al., 2023). A learner may possess reasoning skills without choosing to exercise them consistently. Disposition measures capture the inclination to scrutinise information across situations rather than performance on one task (Sosu, 2013). The Critical Thinking Disposition Scale includes Critical Openness, which concerns receptivity to alternatives and willingness to revise a position, and Reflective Scepticism, which concerns learning from experience and checking the adequacy of reasons and evidence (Sosu, 2013).

Existing evidence does not support a simple conclusion that GenAI either strengthens or weakens critical thought. A quantitative synthesis of 29 experiments and quasi-experiments reported a moderate positive pooled effect of instruction supported by GenAI on higher-order thinking, alongside substantial variation across studies (Zhao et al., 2025). Individual studies illustrate why that variation matters. During a scientific inquiry task, students assigned to ChatGPT reported less cognitive load but produced weaker justifications and argumentation than those using Google (Stadler et al., 2024). In a college writing study, the ChatGPT group showed greater improvement in essay scores, while differences in knowledge gain and transfer across conditions were not statistically significant; process data also documented less frequent engagement in some metacognitive activities (Fan et al., 2025). Within a quasi-experimental study of postgraduate business students, scores aligned with Bloom’s taxonomy increased across several levels in the ChatGPT group, with larger changes generally observed at lower levels. Because the control group did not complete the posttest, those changes cannot confidently be attributed to ChatGPT (Essien et al., 2024). By contrast, structured guidance requiring an initial answer before hints produced more favourable reports of higher-order thinking and self-regulated learning, as well as chemistry learning performance, than unrestricted ChatGPT in a small study (Lee et al., 2024).

Survey findings also point to both enabling and constraining patterns. Among Chinese university students, greater AI dependence was associated with lower levels of critical thinking reported by students, with a significant indirect association through cognitive fatigue (Tian and Zhang, 2025). Another study using the Critical Thinking Disposition Scale identified two opposing indirect associations: general GenAI use was positively related to CTD through perceived confidence in one’s ability and negatively related to CTD through perceived dependence (Wang et al., 2026). Hou et al. (2025a) modelled students’ critical thinking characteristics as predictors of several forms of GenAI reliance during problem solving. Their cross-sectional design did not establish temporal direction.

Student engagement with AI also includes affective, motivational, and readiness dimensions. In music education, specific dimensions of GenAI interactional experience were associated with enjoyment and behavioural intention (Li, 2026). AI readiness and self-efficacy were related to academic performance (Wang and Li, 2024). Perceptions of AI tools were associated with motivation, engagement, creativity, and learning success (Chen, 2025). Readiness and self-efficacy concern perceived capability and preparedness, while usage orientations concern how students organise academic activity with GenAI.

Task performance, perceived improvement, critical thinking skill, higher-order thinking, and CTD are related but distinct outcomes. Evidence about one cannot be transferred directly to another. Likewise, adoption, frequency, duration, and number of applications describe exposure but may conceal differences in how students work with GenAI.

### 2.2 From amount of use to usage orientations

Educational technologies have long been classified by the relationship they establish with learners. Taylor’s (1980) tutor, tool, and tutee framework differentiated instruction provided by technology, technology used to accomplish a task, and technology directed or programmed by the learner. More recent accounts describe GenAI as a tool, tutor, coach, mentor, teammate, simulator, or student (Mollick and Mollick, 2023). These accounts offer conceptual background rather than direct origins for the present measures. They also describe interaction roles, not fixed properties of a platform. The same system may generate an answer, provide guidance, and support iterative development across different academic activities.

A GenAI usage orientation is defined here as a recurring way of organising academic activity with GenAI. The allocation of task work and evaluative responsibility provides a shared organising lens. The framework is nonexhaustive and nonhierarchical. Its orientations are continuous and nonexclusive. A student may delegate one activity, seek guidance in another, and coordinate iteratively with GenAI in a third.

The framework includes Tool, Guidance, and Collaboration because each captures a different arrangement of academic activity. Tool represents delegative task completion and the adoption of AI output with little revision. Guidance represents requests for explanations, examples, clues, or diagnostic assistance while the student retains responsibility for understanding. Collaboration represents the coordination of task information, prompts, and outputs across successive exchanges under student direction. Cognitive offloading informs Tool (Risko and Gilbert, 2016). Instrumental help-seeking informs Guidance (Nelson-Le Gall, 1981). Distributed cognition informs Collaboration (Hollan et al., 2000). Retaining these conceptually distinguishable orientations preserves their functional differences and allows their associations with CTD to be estimated jointly. These definitions reflect the selected items and do not imply a developmental progression. Table 1 details the boundaries and sources.

**Table 1:** Conceptualisation and operationalisation of the three GenAI usage orientations.

Orientation	Conceptual definition	Behaviours represented by the items	Measurement source and contextual adaptation
Tool	Tool represents delegative task completion and the adoption of AI output with little revision.	Direct submission of task requirements, extensive interaction with GenAI, direct use of output, and adoption with little change.	Four items adapted from Hou et al.’s (2025b) Thoughtless Use dimension for academic GenAI use and a behavioural frequency format.
Guidance	Instrumental help-seeking in which GenAI supplies contingent support while the student retains responsibility for understanding.	Requests for explanations, examples, clues, or diagnostic assistance that support continued work on an academic difficulty.	Four items adapted from Chen and Law’s (2025) Instrumental help-seeking measure and extended from digital game learning to academic GenAI use.
Collaboration	Iterative coordination in which GenAI contributions are developed across successive exchanges.	Efficiency, prompt adjustment, provision of task information, extension of output, experimentation with ways of working, and perceived improvement in work or decision quality.	Six items from the Use AI Tools component of Sidra and Mason’s (2026) Collaborative AI Literacy scale, contextualised for academic GenAI use.

The framework is nonexhaustive and nonhierarchical. Orientations are continuous, nonexclusive, and may coexist within the same student. The three item sets formed separate scores and entered the analyses together. Supplementary Table S1 reports the complete item wording. Throughout this study, Tool denotes comparatively delegative GenAI use.

### 2.3 Tool orientation and cognitive offloading

Cognitive offloading occurs when people use an external action or resource to change the information processing demands of a task (Risko and Gilbert, 2016). Notes, calculators, search engines, and collaborators can preserve limited cognitive resources for operations that require greater attention (Dunn and Risko, 2016; Runge et al., 2019). GenAI extends this possibility because it can externalise retrieval, summarising, drafting, reasoning, and solution generation. Its educational significance depends on what the learner delegates and how the returned output is evaluated.

Throughout this study, Tool orientation denotes comparatively delegative use. Its clearest indicators concern direct use of GenAI output and adoption with little revision. The item set was adapted from the Thoughtless Use dimension developed by Hou et al. (2025b). Two items also capture submitting task requirements directly to GenAI and spending substantial time interacting with it. The score therefore represents a broad tendency toward GenAI-centred task completion, with delegation as its conceptual core.

Evaluative judgement requires students to assess the quality of GenAI outputs and the processes used to produce them (Bearman et al., 2024). Greater delegation may coincide with fewer opportunities to compare evidence, formulate reasons, and revise a position. Because GenAI outputs can appear fluent, plausible, and relevant even when unsuitable, they may receive insufficient scrutiny (Bearman et al., 2024; Thompson et al., 2013). Research on automation distinguishes appropriate reliance from misuse, which occurs when an automated aid is trusted despite circumstances that warrant closer supervision (Parasuraman and Riley, 1997). In the GenAI context, Stadler et al. (2024) found that reduced mental effort during scientific inquiry was accompanied by weaker argumentation. A survey of knowledge workers also found that greater confidence in GenAI was associated with less reported enactment of critical thinking, whereas confidence in one’s own task ability was associated with more reported critical engagement (Lee et al., 2025).

The present study assesses the association between Tool orientation and CTD. It does not measure the proposed process or establish temporal order.

### 2.4 Guidance orientation and instrumental help-seeking

Help-seeking can be an adaptive problem-solving strategy when learners obtain the assistance needed to overcome an impasse while preserving responsibility for completing and understanding the task. Nelson-Le Gall (1981) described this as instrumental help-seeking and distinguished it from assistance sought mainly to secure an answer or reduce the learner’s own work. This distinction closely reflects the boundary between Guidance and Tool orientations. Both begin with a request to GenAI, but Guidance is organised around advancing the student’s reasoning through explanations, examples, prompts, or diagnosis.

The Guidance measure draws on Chen and Law’s (2025) Instrumental help-seeking scale. Their study involved middle school students using ChatGPT in a physics game, where instrumental help-seeking was positively related to a combined indicator of game performance and engagement. The present study extends the item context to academic learning while retaining requests for explanation, examples, clues, and diagnostic assistance.

Guidance may be compatible with CTD because a suitable explanation or prompt can keep the learner working on the problem rather than replacing that work (van de Pol et al., 2010). Feedback is most useful when it clarifies the task, the learner’s current position, and productive next steps (Hattie and Timperley, 2007). Its value nevertheless depends on uptake. Students must interpret the information, judge its relevance and quality, and use it to modify subsequent work (Carless and Boud, 2018). Brown et al. (2016) similarly found that students’ active use of feedback was positively associated with self-regulated learning, confidence in their ability, and academic achievement. Receiving information is therefore not sufficient; the learner’s response to it is central.

Evidence from GenAI settings reinforces this distinction. Students who consulted ChatGPT during an essay revision task sometimes skipped evaluation and asked more operational questions, indicating that easy access to conversational support does not automatically produce instrumental help-seeking (Chen et al., 2025). By contrast, Lee et al.'s (2024) system with a guidance mechanism required an initial student answer before providing hints and encouraged iterative revision rather than direct answer retrieval. The guided group reported stronger higher-order thinking and self-regulated learning and achieved better chemistry learning performance than the conventional ChatGPT group. These findings describe Guidance as a pattern in which learners retain problem ownership and engage in explanation, checking, and revision.

### 2.5 Collaboration orientation and iterative coordination

Collaboration involves sustained coordination across student actions and system contributions. Distributed cognition views reasoning as activity organised across people, representations, and artefacts (Hollan et al., 2000). Applied here, this perspective draws attention to task framing, provision of context, revision of prompts, and development of output across successive exchanges.

Sidra and Mason (2026) describe effective collaboration with GenAI as an iterative process organised around explicit goals and human direction. The present measure draws on the Use AI Tools component of their Collaborative AI Literacy scale. The items were contextualised for academic GenAI use. The items cover efficiency, prompt adjustment, provision of task information, experimentation with ways of working, extension of output, and perceived improvement in work or decision quality. Accordingly, Collaboration is treated as iterative coordination under student direction.

Collaboration may be associated with CTD. In academic writing, iterative work with GenAI involved successive prompting, reading, editing, and integration of generated material (Nguyen et al., 2024). Lee et al. (2025) found that knowledge workers reported changes in where they applied critical thinking during GenAI use. These reports concerned verification, integration, and responsibility for the final work. The present items did not measure these cognitive processes. Process data are needed to test this account.

Repeated prompting can occur without careful evaluation. CTD may also help explain differences in these interaction patterns. Hou et al. (2025a) modelled critical thinking characteristics as predictors of reflective, cautious, and collaborative reliance behaviours.

Guidance and Collaboration differ in how support is organised. Guidance addresses a defined learning difficulty through explanations, examples, clues, or diagnostic assistance that allow the student to continue reasoning. Collaboration coordinates task information, prompts, and outputs across successive exchanges toward a broader academic goal. The distinction rests on the function and organisation of the interaction. Frequency and intensity do not define the boundary. Both orientations may coexist across academic activities.

Taken together, cognitive offloading, instrumental help-seeking, and distributed cognition suggest that the three orientations differ in how students organise academic work with GenAI. Because these orientations may coexist, their bivariate associations with CTD may differ from their mutually adjusted associations.

## 3 Methods

### 3.1 Research design

This cross-sectional online survey assessed associations between GenAI usage orientations and CTD among students enrolled in higher education in China. All focal variables were measured at one time point. The analyses therefore estimate bivariate and adjusted associations and do not establish causal effects.

### 3.2 Participants and procedure

The study used nonprobability convenience sampling. Data were collected online through Questionnaire Star and Huixiang Data from May to July 2026. Participants were enrolled in vocational college, undergraduate, or postgraduate programmes and came from 30 provincial-level administrative regions across China. Completion required approximately 5–8 min.

The study was approved by the ethics review committee of Weifang Engineering Vocational College. Participants provided electronic informed consent before accessing the survey and could discontinue participation any time before submission. The survey covered demographic characteristics, GenAI experience, usage orientations, and CTD.

The merged dataset contained 447 responses. Screening covered missing focal items, duplicate identifiers, values outside the permitted range, and uniform responding across the 14 orientation and 11 CTD items. Seventeen respondents selected the same option for every focal item and were excluded. No case was removed for another screening reason. The final analytic sample comprised 430 responses, giving a data-screening retention rate of 96.20%.

### 3.3 Measures

The questionnaire drew on published measures. Fourteen items operationalised Tool, Guidance, and Collaboration as separate GenAI usage orientation scores. The 11 CTD items retained the content of Sosu’s (2013) scale. Orientation items recorded behavioural frequency from 1 (never) to 5 (almost always). CTD items ranged from 1 (not at all true of me) to 5 (very true of me). Table 1 maps each orientation to its definition, represented behaviours, source, and contextual adaptation. Supplementary Table S1 provides the complete item wording.

The orientation items used behavioural frequency anchors, whereas the CTD items asked how true each statement was of the respondent. This difference may reduce common scale and anchor effects (Podsakoff et al., 2003).

The following sections describe each measure, its source, item count, substantive coverage, and scoring.

#### 3.3.1 GenAI usage orientations

Fourteen items represented the three usage orientations (see Supplementary Table S1). Respondents considered their academic GenAI use during the previous 3 months and rated the frequency of each behaviour. The arithmetic mean of the relevant items formed each score; higher values indicate more frequent enactment of that orientation.

The three scores describe how students organise academic activity with GenAI. The allocation of task work and evaluative responsibility provides a shared lens. Tool represents delegative task completion. Guidance covers targeted assistance that retains learner responsibility. Collaboration describes iterative coordination under student direction. This structure supports comparison while preserving the conceptual boundaries of the three orientations.

Tool comprised four items adapted from Hou et al.’s (2025b) Thoughtless Use dimension. Direct use of output and adoption with little revision represent its delegative core; the remaining items assess direct submission of task requirements and extensive interaction with GenAI. Guidance comprised four items adapted from Chen and Law’s (2025) Instrumental help-seeking measure. References to digital game learning were replaced with academic difficulties, while explanations, examples, clues, and diagnostic assistance remained central. Collaboration comprised six items from the Use AI Tools component of Sidra and Mason’s (2026) Collaborative AI Literacy scale. They assess efficiency, prompt adjustment, provision of task information, extension of output, experimentation with ways of working, and perceived improvement in work or decision quality.

The three orientation measures were contextualised for academic GenAI use. The selected item content was retained. Before data collection, the adapted GenAI items were reviewed by three experts. Their expertise covered educational measurement, GenAI in education, and educational psychology. They evaluated item clarity, relevance, conceptual consistency, and suitability for the academic GenAI context. Revisions were made based on their feedback. Content validity was assessed qualitatively through the expert review. Their appraisal supported the final measures.

Contextual changes concerned the academic activity, the 3-month reference period, and the behavioural response format. Because the item sets originated in different conceptual and empirical settings, the combined measurement structure was evaluated through competing CFA models, standardised loadings, reliability, convergent validity, and discriminant validity.

#### 3.3.2 Critical thinking disposition

CTD (see Supplementary Table S1) was measured with Sosu’s (2013) 11-item Critical Thinking Disposition Scale without adaptation to a GenAI context. Seven items represent Critical Openness (CO), including receptivity to new ideas, multiple sources, and alternative viewpoints. Four represent Reflective Scepticism (RS), including source checking and reflection on experience and the wider consequences of decisions.

Sosu (2013) specified a correlated two-factor structure. In this sample, CO and RS showed insufficient empirical separation, while the 11-item one-factor model fitted well and the total score was highly reliable. The mean of all 11 items therefore formed the primary CTD score. This sample-specific decision does not imply that the instrument is universally unidimensional. Original CO and RS means were retained for supplementary analyses.

#### 3.3.3 Covariates

Regression models adjusted for gender, educational level, field of study, duration of GenAI use, weekly use, and self-reported AI proficiency. Reference groups were male respondents, bachelor’s students, humanities or social sciences, more than 1 year of GenAI experience, almost daily use, and intermediate proficiency. Sparse categories were combined as follows: never with less than 3 months; rarely with one or two uses per week; and advanced with expert proficiency.

### 3.4 Data analysis

Analyses were conducted in Stata/MP 19. Screening evaluated missingness, duplicate identifiers, response ranges, and uniform responding. Preliminary diagnostics covered composite score skewness and kurtosis, the first unrotated principal component from Harman’s procedure, variance inflation factors, and heteroskedasticity. Harman’s result was treated as a limited descriptive diagnostic and not as evidence that common method bias was absent.

The five-point items were treated as approximately continuous. Confirmatory factor analysis (CFA) used maximum likelihood estimation with Satorra–Bentler corrections for departures from normality. Fit assessment considered CFI, TLI, RMSEA, SRMR, AIC, and BIC. Information criteria were compared only across models fitted to the same observed items.

Cronbach’s alpha and composite reliability (CR) assessed internal consistency. Standardised loadings and average variance extracted (AVE) informed convergent validity. Discriminant validity was assessed with the Fornell–Larcker criterion (Fornell and Larcker, 1981), latent correlations, and heterotrait–monotrait (HTMT) ratios. Values below 0.85 were evaluated against the conservative criterion proposed by Henseler et al. (2015). Construct decisions considered the full set of evidence.

An additional model combined the Guidance and Collaboration indicators while retaining separate Tool and CTD factors. Its robust fit indices and information criteria were compared with those of the primary four-factor model.

Pearson correlations summarised the primary variables. Hierarchical ordinary least squares regression estimated adjusted associations with CTD. Model 1 included gender, educational level, and field of study. Model 2 added duration of GenAI use, weekly use, and proficiency. Model 3 added Tool, Guidance, and Collaboration. Changes in *R*^2^ and HC3 Wald tests assessed the contribution of each block.

The Breusch–Pagan/Cook–Weisberg test indicated heteroskedasticity, so all regression inferences used HC3 robust standard errors. Models with standardised focal predictors and outcomes yielded comparable *β* coefficients and 95% confidence intervals. The complete model was repeated with CO and RS as supplementary outcomes.

A focused coefficient progression analysis fitted five specifications: Tool alone; Tool with all covariates; Tool with the covariates and Guidance; Tool with the covariates and Collaboration; and the complete model. All specifications used the same 430 participants, scoring rules, covariate coding, reference groups, and HC3 robust standard errors.

For sample description, application use breadth was calculated by summing seven binary indicators of selected GenAI applications. The possible range was 0–7. Field differences were estimated with ordinary least squares regression and HC3 standard errors. Marginal predictions provided unadjusted means and 95% confidence intervals, and Bonferroni adjustment was used for pairwise comparisons. Observed counts were grouped as 0–1, 2, 3, and 4 or more applications for the descriptive figure. This exploratory analysis remained separate from the CTD models. Significance tests for correlations and individual regression coefficients were two-tailed, with *α* = 0.05.

## 4 Results

### 4.1 Data screening and participant characteristics

The merged dataset contained 447 records. Seventeen showed uniform responding across all 25 focal items and were excluded. No record was removed for a duplicate identifier, missing focal item, or value outside the permitted range. The final analytic sample was 430, corresponding to a 96.20% data-screening retention rate. Table 2 reports the screening flow and participant characteristics.

**Table 2:** Data screening and participant characteristics.

Panel/characteristic	Category	*n*	%
Panel A. Data screening			
Raw records		447	100.00
Missing or duplicate ID		0	0.00
Missing focal item(s)		0	0.00
Out-of-range item response		0	0.00
Uniform response across all 25 focal items		17	3.80
Final analytic sample		430	96.20
Panel B. Participant characteristics			
Gender	Male	203	47.21
	Female	227	52.79
Education level	Vocational college	171	39.77
	Bachelor’s	186	43.26
	Postgraduate	73	16.98
Field of study	Humanities/social sciences	106	24.65
	Science/engineering	139	32.33
	Business/management	89	20.70
	Arts	50	11.63
	Other	46	10.70
AI-use duration	Never	1	0.23
	<3 months	25	5.81
	3–6 months	55	12.79
	6–12 months	115	26.74
	>1 year	234	54.42
Weekly AI use	Rarely	3	0.70
	1–2 times/week	93	21.63
	3–5 times/week	160	37.21
	Almost daily	174	40.47
AI proficiency	Beginner	76	17.67
	Intermediate	267	62.09
	Advanced	82	19.07
	Expert	5	1.16

Panel A percentages use the raw sample (*N* = 447); exclusion reasons were mutually exclusive. Panel B percentages use the final analytic sample (*N* = 430) and may not total exactly 100% because of rounding.

Respondents selected a mean of 2.80 GenAI applications (SD = 1.16, median = 3, observed range = 0–6). Supplementary Figure S1 shows application prevalence. Breadth differed across fields in the unadjusted model, *F*(4, 425) = 5.53, *p* < 0.001, *R*^2^ = 0.044. After Bonferroni adjustment, the heterogeneous Other category reported lower breadth than humanities or social sciences (*p* = 0.009), science or engineering (*p* < 0.001), and business or management (*p* = 0.002). The difference from Arts was nonsignificant (*p* = 0.111), and no other comparison reached significance. Supplementary Figure S2 shows the within-field composition.

### 4.2 Preliminary analyses

All retained cases had complete data for focal items, composite scores, and regression variables; no imputation was needed. Composite score skewness ranged from −0.765 for Collaboration to −0.117 for Tool, and kurtosis ranged from 2.422 to 3.127. These values showed no severe univariate departure. Table 3 summarises the preliminary diagnostics.

**Table 3:** Summary of preliminary diagnostics.

Diagnostic	Verified result	Analytical implication
Missing values in analytic variables	0	No imputation
Composite-score skewness	−0.765 to −0.117	No severe univariate departure
Composite-score kurtosis	2.422–3.127	No severe univariate departure
Harman first-component variance	38.02%	Descriptive diagnostic only; cannot establish the absence of common method bias
25-item one-factor CFA	CFI = 0.648; TLI = 0.616; RMSEA = 0.125; SRMR = 0.146	Poor fit; inconsistent with a single-factor account
Variance inflation factors	1.15–2.54; mean = 1.54	No problematic multicollinearity
Breusch–Pagan/Cook–Weisberg	*χ*^2^(1) = 11.24, *p* < 0.001	HC3 standard errors used

Harman’s diagnostic and the one-factor CFA cannot establish that common method bias is absent. HC3 standard errors address heteroskedasticity.

The first unrotated principal component in Harman’s diagnostic explained 38.02% of total item variance. The 25-item one-factor CFA fitted poorly (CFI = 0.648, TLI = 0.616, RMSEA = 0.125, SRMR = 0.146). These results do not establish the absence of common method bias. VIFs in the fully adjusted model ranged from 1.15 to 2.54 (mean = 1.54). The Breusch–Pagan/Cook–Weisberg test rejected constant error variance, *χ*^2^(1) = 11.24, *p* < 0.001, supporting the use of HC3 standard errors.

### 4.3 Measurement model

#### 4.3.1 Competing measurement models

Table 4 compares seven CFA specifications. The 25-item one-factor model fitted poorly, as did the model combining the three GenAI orientations while retaining separate CO and RS factors. The original five-factor model and the four-factor model with one CTD factor both fitted well. The five-factor model produced CFI = 0.972, TLI = 0.968, RMSEA = 0.036, and SRMR = 0.055; corresponding values for the four-factor model were 0.969, 0.965, 0.038, and 0.055.

**Table 4:** Comparison of competing measurement models.

Model	*χ*^2^SB	df	*p*	CFI	TLI	RMSEA	SRMR	AIC	BIC
A1.25-item one-factor CFA	2117.386	275	<0.001	0.648	0.616	0.125	0.146	23,603.13	23,907.92
A2.AI orientations combined + CO + RS	1140.117	272	<0.001	0.834	0.817	0.086	0.103	22,417.20	22,734.18
A3.Original five-factor model	410.677	265	<0.001	0.972	0.968	0.036	0.055	21,543.14	21,888.56
A4.Four-factor model with CTD	431.987	269	<0.001	0.969	0.965	0.038	0.055	21,560.08	21,889.24
A5.Tool, combined Guidance and Collaboration, and CTD	615.617	272	<0.001	0.934	0.928	0.054	0.063	21,780.31	22,097.28
B1.CTD one-factor model	80.270	44	<0.001	0.985	0.981	0.044	0.025	8,431.36	8,565.46
B2.CO–RS correlated two-factor model	69.838	43	0.006	0.989	0.986	0.038	0.023	8,418.43	8,556.60

SB, Satorra–Bentler robust statistic. AIC and BIC are compared within the 25-item set (Models A1–A5) or within the 11-item CTD set (Models B1–B2), not across item sets.

The five-factor model had a lower AIC than the four-factor model (ΔAIC = 16.94), whereas the difference in BIC was negligible (ΔBIC = 0.68). Differences in CFI (0.003) and RMSEA (0.002) were also small. Within the 11 CTD items, the correlated two-factor model had lower AIC and BIC than the one-factor model (ΔAIC = 12.93; ΔBIC = 8.86). The one-factor solution nevertheless showed good fit (CFI = 0.985, TLI = 0.981, RMSEA = 0.044, SRMR = 0.025). The scoring decision therefore considered model fit together with reliability and discriminant validity.

Model A5 combined the Guidance and Collaboration indicators while retaining separate Tool and CTD factors. All 10 indicators loaded significantly on the common factor, with standardised loadings from 0.587 to 0.755. Model A5 fitted the data less well than the primary four-factor model. Its CFI and TLI were 0.934 and 0.928, compared with 0.969 and 0.965 for Model A4. RMSEA increased from 0.038 to 0.054, and SRMR increased from 0.055 to 0.063. AIC increased from 21,560.08 to 21,780.31, and BIC increased from 21,889.24 to 22,097.28.

#### 4.3.2 Reliability and convergent validity

Table 5 reports reliability and convergent validity. Standardised loadings ranged from 0.693 to 0.827 for Tool, 0.701 to 0.822 for Guidance, 0.696 to 0.788 for Collaboration, and 0.723 to 0.814 for CTD. Corresponding ranges were 0.727–0.797 for CO and 0.778–0.825 for RS. Across the six scores, Cronbach’s alpha ranged from 0.839 to 0.943, CR from 0.841 to 0.943, and AVE from 0.540 to 0.649. Supplementary Table S2 provides all item loadings.

**Table 5:** Reliability and convergent validity.

Construct	Items	Loading range	*α*	CR	AVE
Tool orientation	4	0.693–0.827	0.851	0.853	0.593
Guidance orientation	4	0.701–0.822	0.839	0.841	0.570
Collaboration orientation	6	0.696–0.788	0.875	0.875	0.540
CTD	11	0.723–0.814	0.943	0.943	0.602
Critical openness (CO)	7	0.727–0.797	0.910	0.911	0.594
Reflective scepticism (RS)	4	0.778–0.825	0.880	0.881	0.649

Tool, Guidance, Collaboration, and CTD loadings and validity estimates come from the primary four-factor model. CO and RS loadings and validity estimates come from the original five-factor model.

#### 4.3.3 Discriminant validity and the CTD decision

The primary four-factor model supported separation among Tool, Guidance, Collaboration, and CTD. The square root of AVE was 0.755 for Guidance and 0.735 for Collaboration, and their latent correlation was 0.731. The Fornell–Larcker margin was therefore small for Collaboration. Their HTMT ratio was 0.728, below the prespecified 0.85 threshold. Other HTMT ratios in the primary model ranged from 0.134 to 0.579. Model A5 provided a direct test of whether Guidance and Collaboration could be represented by one factor. Its weaker fit and higher information criteria support retaining two correlated factors with substantial shared variance.

CO and RS each showed good reliability and convergent validity when considered separately. CO yielded *α* = 0.910, CR = 0.911, and AVE = 0.594; RS yielded *α* = 0.880, CR = 0.881, and AVE = 0.649. Their separation was inadequate in this sample. The latent correlation was 0.963 and exceeded both square roots of AVE (0.771 and 0.806), while HTMT was 0.964. Thus, the lower information criteria of the correlated two-factor solution did not establish practically distinct subscales. Given the good fit and high reliability of the 11-item one-factor solution, CTD was retained as the primary outcome. Table 6 summarises the discriminant validity decision, and Supplementary Table S3 presents the full Fornell–Larcker and HTMT matrices.

**Table 6:** Discriminant validity summary.

Construct pair	Latent *r*	HTMT	√AVE values	Decision at HTMT < 0.85
Tool–Guidance	0.320	0.322	0.770/0.755	Supported
Tool–Collaboration	0.285	0.303	0.770/0.735	Supported
Tool–CTD	−0.060	0.134	0.770/0.776	Supported
Guidance–Collaboration	0.731	0.728	0.755/0.735	Supported; marginal Fornell–Larcker separation
Guidance–CTD	0.427	0.432	0.755/0.776	Supported
Collaboration–CTD	0.573	0.579	0.735/0.776	Supported
CO–RS	0.963	0.964	0.771/0.806	Not supported

Primary model values come from the four-factor CFA; CO and RS values come from the original five-factor CFA. Discriminant validity was evaluated by comparing each square root of AVE with the absolute latent correlation and by applying the 0.85 HTMT criterion. Full matrices appear in Supplementary Table S3.

### 4.4 Descriptive statistics and correlations

Table 7 summarises the primary variables to answer RQ1. Specifically, Tool correlated positively with Guidance (*r* = 0.266, *p* < 0.001) and Collaboration (*r* = 0.254, *p* < 0.001), but its zero-order association with CTD was small and nonsignificant (*r* = −0.044, *p* = 0.363). Guidance (*r* = 0.384, *p* < 0.001) and Collaboration (*r* = 0.525, *p* < 0.001) were positively related to CTD. Guidance and Collaboration were also positively correlated (*r* = 0.622, *p* < 0.001).

**Table 7:** Descriptive statistics and zero-order correlations among primary variables.

Variable	*M*	SD	1	2	3	4
1.Tool orientation	3.051	0.870	–			
2.Guidance orientation	3.855	0.715	0.266***	–		
3.Collaboration orientation	3.870	0.676	0.254***	0.622***	–	
4.CTD	3.905	0.654	−0.044	0.384***	0.525***	–

*N* = 430. Values below the diagonal are two-tailed Pearson correlations. ***p < 0.001.

Correlations involving CO and RS appear in Supplementary Table S4.

### 4.5 Hierarchical regression predicting CTD

Next, a hierarchical regression was conducted to answer RQ2 (see Table 8). The demographic block explained 23.69% of CTD variance (*R*^2^ = 0.237, adjusted *R*^2^ = 0.224, *F*(7, 422) = 20.06, *p* < 0.001). Adding duration, weekly use, and proficiency increased *R*^2^ by 0.049 (block Wald *F* = 2.60, *p* = 0.012). The orientation block added 0.104 in Model 3 (block Wald *F* = 22.34, *p* < 0.001). The final model explained 38.95% of CTD variance (*R*^2^ = 0.390, adjusted *R*^2^ = 0.364, *F*(17, 412) = 22.07, *p* < 0.001).

**Table 8:** Hierarchical regression predicting CTD.

Panel A. Model summary								
Model	*N*	*R* ^2^	Adj. *R*^2^	Δ*R*^2^	Model *F*	*p*	Block *F*	*p*
1. Demographics	430	0.237	0.224	–	20.06	<0.001	–	–
2. + AI-use background	430	0.286	0.262	0.049	11.90	<0.001	2.60	0.012
3. + Usage orientations	430	0.390	0.364	0.104	22.07	<0.001	22.34	<0.001

Panel B. Focal predictors in Model 3					
Predictor	*B*	HC3 SE	*β*	95% CI for *β*	*p*
Tool orientation	−0.142	0.032	−0.189	[−0.274, −0.104]	<0.001
Guidance orientation	0.115	0.048	0.126	[0.023, 0.229]	0.017
Collaboration orientation	0.311	0.080	0.321	[0.159, 0.483]	<0.001

Model 1 included gender, education, and field; Model 2 added duration of AI use, weekly use, and proficiency; Model 3 added the three GenAI usage orientations. Panel B confidence intervals come from models with standardised focal predictors and outcome. All inferences use HC3 robust standard errors. Complete coefficients appear in Supplementary Table S6.

Tool was negatively associated with CTD in the complete model (*B* = −0.142, HC3 SE = 0.032, *β* = −0.189, 95% CI for *β* [−0.274, −0.104], *p* < 0.001). Supplementary Table S5 traces the coefficient across five specifications. Tool alone produced a near zero estimate (*β* = −0.044, 95% CI [−0.151, 0.063], *p* = 0.421). Adding all covariates produced the first clear negative estimate (*β* = −0.108, 95% CI [−0.202, −0.014], *p* = 0.024). The coefficient was −0.163 when Guidance was added and −0.176 when Collaboration was added. It reached −0.189 in the complete model. The estimate was negative in every adjusted specification. This progression is consistent with statistical suppression involving covariate adjustment and shared variance among the orientations (MacKinnon et al., 2000). The complete model coefficient represents a conditional association.

Guidance was positively associated with CTD (*B* = 0.115, HC3 SE = 0.048, *β* = 0.126, 95% CI for *β* [0.023, 0.229], *p* = 0.017). Collaboration also showed a positive association (*B* = 0.311, HC3 SE = 0.080, *β* = 0.321, 95% CI for *β* [0.159, 0.483], *p* < 0.001). Collaboration had the largest standardised point estimate, but no formal test compared its coefficient with the Guidance coefficient.

Among the covariates, female respondents reported lower CTD than male respondents in Model 3 (*B* = −0.213, HC3 SE = 0.059, 95% CI [−0.329, −0.097], *p* < 0.001). Educational level and field contrasts did not reach 0.05 after the orientations entered the model. Beginner proficiency approached but did not reach significance (*B* = −0.167, *p* = 0.056); other GenAI background contrasts were nonsignificant. Supplementary Table S6 reports all coefficients.

### 4.6 Supplementary CTD subscale analyses

The fully adjusted model was repeated with CO and RS as supplementary outcomes. Tool remained negative for CO (*β* = −0.164, 95% CI [−0.250, −0.078], *p* < 0.001) and RS (*β* = −0.215, 95% CI [−0.302, −0.128], *p* < 0.001). Collaboration remained positive for CO (*β* = 0.334, 95% CI [0.173, 0.495], *p* < 0.001) and RS (*β* = 0.273, 95% CI [0.110, 0.436], *p* = 0.001). Guidance was positive in both models and reached conventional significance for RS (*β* = 0.174, *p* = 0.003), but not CO (*β* = 0.090, *p* = 0.079).

Figure 1 places the fully adjusted coefficients for CTD, CO, and RS on a common standardised metric. Tool was negative and Collaboration positive across all outcomes. The Guidance estimate was smaller and less consistent across the subscales. CO and RS form part of CTD and were highly correlated in this sample. These models provide supplementary detail. Numerical estimates appear in Supplementary Table S7.

**Figure 1:**
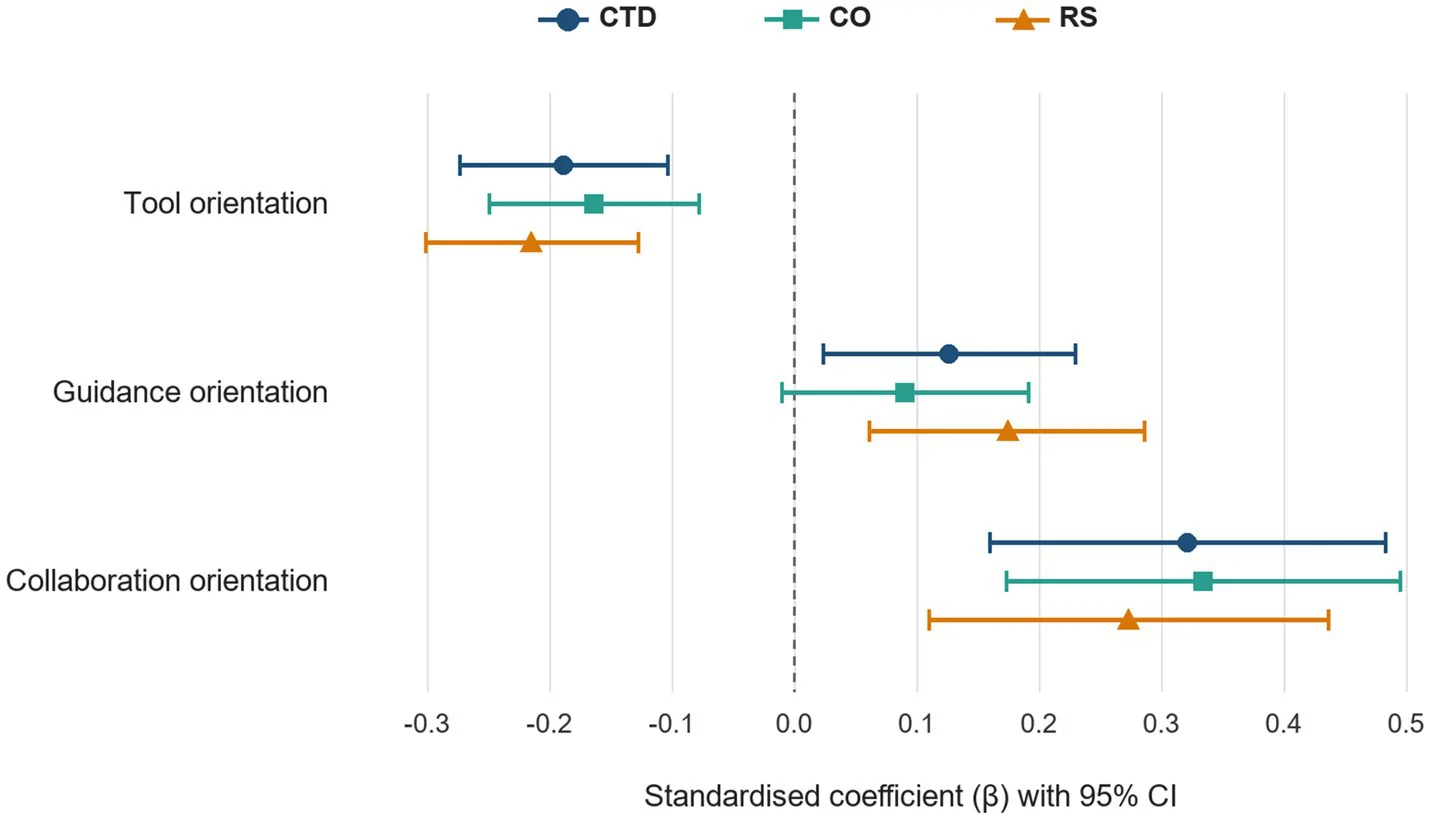
Standardised associations of GenAI usage orientations with CTD, CO, and RS. Points are standardised coefficients from fully adjusted models; horizontal lines are 95% confidence intervals calculated with HC3 robust standard errors. Each model adjusted for gender, educational level, field of study, duration of GenAI use, weekly use, and AI proficiency. CTD was the primary outcome. CO and RS were supplementary outcomes from the original subscale scoring of the same 11 items.

## 5 Discussion

This study assessed whether Tool, Guidance, and Collaboration orientations were differently related to CTD. Guidance and Collaboration had positive bivariate associations, while Tool had a near-zero correlation. After adjustment, Tool was negative and the other two orientations remained positive. Their addition accounted for 10.37% of additional CTD variance beyond demographic characteristics and general GenAI experience.

The Tool result requires an adjusted interpretation. Tool correlated positively with Guidance and Collaboration, yet its adjusted association with CTD was negative. The change from *r* = −0.044 to *β* = −0.189 is consistent with statistical suppression. Students may delegate work in some activities and seek active support in others. At comparable levels of the other orientations and covariates, a higher Tool score indicates comparatively greater reliance on GenAI-centred task completion. It does not imply a simple negative relationship between Tool and CTD.

Cognitive offloading offers one possible account of the adjusted Tool association. External resources can reduce task demands and free capacity for other operations (Risko and Gilbert, 2016). Evaluative judgement requires students to assess the quality of GenAI outputs and the processes used to produce them (Bearman et al., 2024). On this account, greater delegation may coincide with fewer opportunities for evidence comparison, reason formulation, and revision. Stadler et al. (2024) found that reduced effort during scientific inquiry coincided with weaker justification and argumentation. Lee et al. (2025) reported an association between greater confidence in GenAI and less reported critical thinking effort among knowledge workers. These studies provide possible explanations for the adjusted Tool coefficient. The present data do not identify the process operating in this sample.

Guidance retained a modest positive association with CTD. This result aligns with instrumental help-seeking, in which assistance helps a learner move beyond an impasse while responsibility for understanding remains with the learner (Nelson-Le Gall, 1981). Such support depends on how students interpret and use explanations or feedback (Carless and Boud, 2018). Chen and Law (2025) related instrumental help-seeking from ChatGPT to a combined indicator of game performance and engagement. Lee et al. (2024) also reported favourable outcomes when students provided an initial answer before receiving hints. Guidance may therefore accompany critical engagement when assistance informs continued student reasoning.

Collaboration had the largest positive standardised point estimate and remained positive in the CO and RS models. Its items cover provision of task information, prompt adjustment, experimentation, extension of output, efficiency, and perceived work quality. Distributed cognition offers a useful account of such coordination because activity can develop across people, representations, and technological resources (Hollan et al., 2000). The positive adjusted association for Collaboration is compatible with comparison and revision during iterative exchanges. These cognitive processes were not measured directly. The point estimate does not show that Collaboration was significantly more strongly related to CTD than Guidance because the coefficients were not formally compared.

The supplementary models broadly reflected the primary CTD pattern: Tool remained negative and Collaboration positive for both CO and RS, whereas Guidance showed positive estimates for both subscales, with a weaker pattern for CO.

### 5.1 Theoretical implications

The framework contributes to educational psychology by placing the organisation of academic activity at the centre of analysis. AI reliance models describe students’ responses to AI output (Hou et al., 2025b). AI literacy models focus on the knowledge and capabilities required for effective use (Sidra and Mason, 2026). Prior research has also linked critical thinking characteristics with several reliance behaviours (Hou et al., 2025a). The present study complements this work by treating CTD as the statistical outcome and estimating Tool, Guidance, and Collaboration together across general academic activities. This comparative account draws on cognitive offloading, instrumental help-seeking, and distributed cognition. The allocation of task work and evaluative responsibility provides a shared organising lens.

The results show the analytical value of these distinctions. When the orientation block was entered after demographic characteristics and general GenAI experience, *R*^2^ increased by 0.1037, or 10.37 percentage points. The full model showed a negative adjusted association between Tool and CTD and positive adjusted associations for Guidance and Collaboration. These findings indicate that the organisation of GenAI use provides information about CTD beyond broad measures of experience.

The contrast between the bivariate and adjusted Tool estimates further shows why correlated orientations should be modelled together. Aggregate use scores and isolated correlations can conceal such conditional patterns. Future process studies can measure task work and evaluative responsibility directly.

Guidance and Collaboration were strongly related. The combined factor model fitted worse than the model with separate factors, supporting two conceptually distinguishable, strongly correlated orientations. With only two first-order factors, a conventional second-order model requires additional identifying constraints and adds little beyond their covariance. It cannot test progression from Guidance to Collaboration. Longitudinal and task process data are needed to assess that sequence.

### 5.2 Practical implications

Course design can require students to document their reasoning and evaluation during GenAI use. Teachers may ask students to draft an initial response before requesting explanations, examples, clues, or diagnostic assistance. Assignments may require comparison of AI outputs, source checks, and reasons for retaining or revising material. Course instructions may also distinguish routine assistance from delegation of core analysis. These options foreground the evaluation of AI outputs and processes (Bearman et al., 2024).

Teachers in Chinese university music classes associated scaffolding with learner autonomy and collaboration. They also identified diverse learner needs and support timing as practical challenges (Li and Wang, 2026).

The present study does not establish the effects of these teaching practices on CTD. Intervention studies should test these practices using CTD measures and direct critical thinking tasks.

### 5.3 Limitations and future research

The cross-sectional design prevents temporal inference. The models cannot determine whether orientation patterns precede later CTD or whether existing CTD shapes how strongly students engage in each orientation. Unmeasured motivation, prior achievement, course design, and institutional context may relate to both. Longitudinal and experience sampling designs could trace temporal patterns and changes within students. Experiments could test causal effects across comparable forms of GenAI support.

The nonprobability convenience sample covered 30 provincial administrative regions but does not represent all Chinese college students. Beyond the factors noted above, other unmeasured variables include course type, teacher AI policies, institutional AI rules, digital literacy, and prior training in critical thinking. These factors may relate to both orientation scores and CTD. The adjusted coefficients are conditional on the variables included in the models.

All focal variables came from one self-report questionnaire administered at one time point. Harman’s single-factor test provides only a limited descriptive diagnostic and cannot establish the absence of common method bias. Shared method variance may affect the estimated associations. Future studies should separate predictor and outcome measurement in time and draw on multiple data sources (Podsakoff et al., 2003, 2012), for example by combining surveys with GenAI conversation records, coded measures of prompt quality, output revision traces, source evaluation tasks, direct critical reasoning tasks, and independent evaluations of learning products.

CTD captures disposition and may relate differently to direct reasoning performance. Because CO and RS lacked adequate empirical separation in this sample, the supplementary analyses describe the original subscales and do not constitute independent replication or grounds for revising Sosu’s (2013) model. The orientation measures were drawn from different settings and contextualised for academic GenAI use, so their conceptual boundaries require continued evaluation. Further validation should quantify expert agreement and evaluate item coverage across a wider range of academic settings.

Independent samples should reassess the distinction between CO and RS. Measures of offloading, feedback uptake, and verification could test the proposed processes. Future studies should include more diverse samples from other countries to examine whether GenAI usage orientations and their associations with CTD vary across national contexts.

## 6 Conclusion

This study distinguishes three GenAI usage orientations: Tool, Guidance, and Collaboration, and assesses their associations with CTD. Tool showed no statistically significant bivariate association with CTD and had a negative adjusted coefficient. Guidance and Collaboration showed positive bivariate and adjusted associations. The orientation block increased *R*^2^ by 0.1037, or 10.37 percentage points, beyond demographic characteristics and general GenAI experience. These findings draw attention to how students organise academic activity with GenAI. They reflect associations observed at one time point and do not establish temporal order or causation. Longitudinal, experimental, and process evidence is needed to assess direction, causal effects, and the mechanisms involved.

## Data Availability

The raw data supporting the conclusions of this article will be made available by the authors, without undue reservation.

## References

[ref1] Bastani H. Bastani O. Sungu A. Ge H. Kabakcı Ö. Mariman R. (2025). Generative AI without guardrails can harm learning: evidence from high school mathematics. Proc. Natl. Acad. Sci. USA 122:e2422633122. doi: 10.1073/pnas.2422633122, 40560616 PMC12232635

[ref2] Bearman M. Tai J. Dawson P. Boud D. Ajjawi R. (2024). Developing evaluative judgement for a time of generative artificial intelligence. Assess. Eval. High. Educ. 49, 893–905. doi: 10.1080/02602938.2024.2335321

[ref3] Brown G. T. L. Peterson E. R. Yao E. S. (2016). Student conceptions of feedback: impact on self-regulation, self-efficacy, and academic achievement. Br. J. Educ. Psychol. 86, 606–629. doi: 10.1111/bjep.12126, 27612004

[ref4] Carless D. Boud D. (2018). The development of student feedback literacy: enabling uptake of feedback. Assess. Eval. High. Educ. 43, 1315–1325. doi: 10.1080/02602938.2018.1463354

[ref5] Chan C. K. Y. Hu W. (2023). Students’ voices on generative AI: perceptions, benefits, and challenges in higher education. Int. J. Educ. Technol. High. Educ. 20:43. doi: 10.1186/s41239-023-00411-8

[ref6] Chen L. (2025). Unlocking the beat: how AI tools drive music students’ motivation, engagement, creativity and learning success. Eur. J. Educ. 60:e12823. doi: 10.1111/ejed.12823

[ref7] Chen C.-H. Law V. (2025). The role of help-seeking from ChatGPT in digital game-based learning. Educ. Technol. Res. Dev. 73, 1703–1721. doi: 10.1007/s11423-025-10464-3

[ref8] Chen A. Xiang M. Zhou J. Jia J. Shang J. Li X. . (2025). Unpacking help-seeking process through multimodal learning analytics: a comparative study of ChatGPT vs human expert. Comput. Educ. 226:105198. doi: 10.1016/j.compedu.2024.105198

[ref9] Dunn T. L. Risko E. F. (2016). Toward a metacognitive account of cognitive off-loading. Cogn. Sci. 40, 1080–1127. doi: 10.1111/cogs.12273 26306555

[ref10] Essien A. Bukoye O. T. O’Dea X. Kremantzis M. (2024). The influence of AI text generators on critical thinking skills in UK business schools. Stud. High. Educ. 49, 865–882. doi: 10.1080/03075079.2024.2316881

[ref11] Fan Y. Tang L. Le H. Shen K. Tan S. Zhao Y. . (2025). Beware of metacognitive laziness: effects of generative artificial intelligence on learning motivation, processes, and performance. Br. J. Educ. Technol. 56, 489–530. doi: 10.1111/bjet.13544

[ref12] Fornell C. Larcker D. F. (1981). Evaluating structural equation models with unobservable variables and measurement error. J. Mark. Res. 18, 39–50. doi: 10.2307/3151312

[ref13] Halpern D. F. (1998). Teaching critical thinking for transfer across domains: disposition, skills, structure training, and metacognitive monitoring. Am. Psychol. 53, 449–455. doi: 10.1037/0003-066X.53.4.449 9572008

[ref15] Hattie J. Timperley H. (2007). The power of feedback. Rev. Educ. Res. 77, 81–112. doi: 10.3102/003465430298487

[ref16] Henseler J. Ringle C. M. Sarstedt M. (2015). A new criterion for assessing discriminant validity in variance-based structural equation modeling. J. Acad. Mark. Sci. 43, 115–135. doi: 10.1007/s11747-014-0403-8

[ref17] Hollan J. Hutchins E. Kirsh D. (2000). Distributed cognition: toward a new foundation for human–computer interaction research. ACM Trans. Comput. Hum. Interact. 7, 174–196. doi: 10.1145/353485.353487

[ref18] Hou C. Zhu G. Sudarshan V. (2025a). The role of critical thinking on undergraduates’ reliance behaviours on generative AI in problem-solving. Br. J. Educ. Technol. 56, 1919–1941. doi: 10.1111/bjet.13613

[ref19] Hou C. Zhu G. Sudarshan V. Lim F. S. Ong Y. S. (2025b). Measuring undergraduate students’ reliance on generative AI during problem-solving: scale development and validation. Comput. Educ. 234:105329. doi: 10.1016/j.compedu.2025.105329

[ref20] Johnston H. Wells R. F. Shanks E. M. Boey T. Parsons B. N. (2024). Student perspectives on the use of generative artificial intelligence technologies in higher education. Int. J. Educ. Integr. 20:2. doi: 10.1007/s40979-024-00149-4

[ref21] Lee H.-Y. Chen P.-H. Wang W.-S. Huang Y.-M. Wu T.-T. (2024). Empowering ChatGPT with guidance mechanism in blended learning: effect of self-regulated learning, higher-order thinking skills, and knowledge construction. Int. J. Educ. Technol. High. Educ. 21:16. doi: 10.1186/s41239-024-00447-4

[ref22] Lee H.-P. Sarkar A. Tankelevitch L. Drosos I. Rintel S. Banks R. . (2025). The impact of generative AI on critical thinking: self-reported reductions in cognitive effort and confidence effects from a survey of knowledge workers. in Proceedings of the 2025 CHI Conference on Human Factors in Computing Systems. Article 1121, pp. 1–22. Association for Computing Machinery. doi: 10.1145/3706598.3713778

[ref23] Li P. (2026). Music education with GenAI: exploring the mediating roles of enjoyment between smart service interactional experience and behavioural intention. Eur. J. Educ. 61:e70423. doi: 10.1111/ejed.70423

[ref24] Li P. Wang Y. (2026). Favourable outcomes, challenges and discipline-specific implications of teacher scaffolding in Chinese university music classes: a qualitative study. Eur. J. Educ. 61:e70568. doi: 10.1111/ejed.70568

[ref25] Loyens S. M. M. van Meerten J. E. Schaap L. Wijnia L. (2023). Situating higher-order, critical, and critical-analytic thinking in problem- and project-based learning environments: a systematic review. Educ. Psychol. Rev. 35:39. doi: 10.1007/s10648-023-09757-x, 30311153

[ref26] MacKinnon D. P. Krull J. L. Lockwood C. M. (2000). Equivalence of the mediation, confounding and suppression effect. Prev. Sci. 1, 173–181. doi: 10.1023/A:1026595011371, 11523746 PMC2819361

[ref27] Miao F. Holmes W. (2023). Guidance for Generative AI in Education and Research. Paris: UNESCO. doi: 10.54675/EWZM9535

[ref28] Mollick E. R. Mollick L. (2023). Assigning AI: Seven Approaches for Students, with Prompts. The Wharton School Research Paper. SSRN. doi: 10.2139/ssrn.4475995

[ref29] Nelson-Le Gall S. (1981). Help-seeking: an understudied problem-solving skill in children. Dev. Rev. 1, 224–246. doi: 10.1016/0273-2297(81)90019-8

[ref30] Nguyen A. Hong Y. Dang B. Huang X. (2024). Human–AI collaboration patterns in AI-assisted academic writing. Stud. High. Educ. 49, 847–864. doi: 10.1080/03075079.2024.2323593

[ref31] Parasuraman R. Riley V. (1997). Humans and automation: use, misuse, disuse, abuse. Hum. Factors 39, 230–253. doi: 10.1518/001872097778543886

[ref32] Podsakoff P. M. MacKenzie S. B. Lee J.-Y. Podsakoff N. P. (2003). Common method biases in behavioral research: a critical review of the literature and recommended remedies. J. Appl. Psychol. 88, 879–903. doi: 10.1037/0021-9010.88.5.879, 14516251

[ref33] Podsakoff P. M. MacKenzie S. B. Podsakoff N. P. (2012). Sources of method bias in social science research and recommendations on how to control it. Annu. Rev. Psychol. 63, 539–569. doi: 10.1146/annurev-psych-120710-100452 21838546

[ref34] Risko E. F. Gilbert S. J. (2016). Cognitive offloading. Trends Cogn. Sci. 20, 676–688. doi: 10.1016/j.tics.2016.07.002 27542527

[ref35] Runge Y. Frings C. Tempel T. (2019). Saving-enhanced performance: saving items after study boosts performance in subsequent cognitively demanding tasks. Memory 27, 1462–1467. doi: 10.1080/09658211.2019.1654520, 31418314

[ref36] Sidra S. Mason C. (2026). Generative AI in human–AI collaboration: validation of the collaborative AI literacy and collaborative AI metacognition scales for effective use. Int. J. Hum. Comput. Interact. 42, 5084–5108. doi: 10.1080/10447318.2025.2543997

[ref37] Sosu E. M. (2013). The development and psychometric validation of a critical thinking disposition scale. Think. Skills Creat. 9, 107–119. doi: 10.1016/j.tsc.2012.09.002

[ref38] Stadler M. Bannert M. Sailer M. (2024). Cognitive ease at a cost: LLMs reduce mental effort but compromise depth in student scientific inquiry. Comput. Hum. Behav. 160:Article 108386. doi: 10.1016/j.chb.2024.108386

[ref39] Suriano R. Plebe A. Acciai A. Fabio R. A. (2025). Student interaction with ChatGPT can promote complex critical thinking skills. Learn. Instr. 95:Article 102011. doi: 10.1016/j.learninstruc.2024.102011

[ref40] Taylor R. P. (ed.) (1980). The Computer in the School: Tutor, Tool, Tutee. New York, NY: Teachers College Press.

[ref41] Thompson V. A. Prowse Turner J. A. Pennycook G. Ball L. J. Brack H. Ophir Y. . (2013). The role of answer fluency and perceptual fluency as metacognitive cues for initiating analytic thinking. Cognition 128, 237–251. doi: 10.1016/j.cognition.2012.09.012 23158572

[ref42] Tian J. Zhang R. (2025). Learners’ AI dependence and critical thinking: the psychological mechanism of fatigue and the social buffering role of AI literacy. Acta Psychol. 260:105725. doi: 10.1016/j.actpsy.2025.105725 41076923

[ref43] van de Pol J. Volman M. Beishuizen J. (2010). Scaffolding in teacher–student interaction: a decade of research. Educ. Psychol. Rev. 22, 271–296. doi: 10.1007/s10648-010-9127-6

[ref44] Wang X. Li P. (2024). Assessment of the relationship between music students’ self-efficacy, academic performance and their artificial intelligence readiness. Eur. J. Educ. 59:e12761. doi: 10.1111/ejed.12761

[ref45] Wang Z. Li W. Liu F. Yao J. Wang Y. (2026). Enhancing or eroding? Exploring how generative artificial intelligence usage influences college students’ critical thinking disposition. Curr. Psychol. 45:1033. doi: 10.1007/s12144-026-09589-2

[ref46] Zhao Y. Yue Y. Sun Z. Jiang Q. Li G. (2025). Does generative artificial intelligence improve students’ higher-order thinking? A meta-analysis based on 29 experiments and quasi-experiments. J. Intelligence 13:160. doi: 10.3390/jintelligence13120160, 41440821 PMC12734368

