# Supplementary material for: Associations between generative AI usage orientations and critical thinking disposition among college students in China
Source: Front Psychol. 2026 Sep 28;17:1957029. doi: 10.3389/fpsyg.2026.1957029 (PMC13685548; doi:10.3389/fpsyg.2026.1957029)
Supplement: Supplementary file 1 [file Data_Sheet_1.pdf]

Supplementary Materials

The supplementary materials present the scale sources and complete wording of all 25 questionnaire items, GenAI application use patterns, measurement properties, correlations, and regression analyses for CTD and its original subscales.

Table S1.

Scale sources and complete item wording

Construct and source	Item	Final item wording
Tool orientation (Hou et al., 2025b)	T1	When completing academic tasks, I copy the task requirements or problem statements directly to generative AI for help.
Tool orientation (Hou et al., 2025b)	T2	I use generative AI's output directly as part of my coursework or task product.
Tool orientation (Hou et al., 2025b)	T3	When completing academic tasks, I spend most of the time interacting with generative AI.
Tool orientation (Hou et al., 2025b)	T4	I adopt generative AI's output without major changes.
Guidance orientation (Chen and Law, 2025)	G1	When I encounter difficulties in my studies, I ask generative AI to explain the relevant knowledge or underlying principles to me.
Guidance orientation (Chen and Law, 2025)	G2	When I encounter difficulties in my studies, I ask generative AI to help me understand by providing examples.
Guidance orientation (Chen and Law, 2025)	G3	When I encounter difficulties in my studies, I ask generative AI to provide prompts or clues that encourage me to think further.
Guidance orientation (Chen and Law, 2025)	G4	When I encounter difficulties in my studies, I ask generative AI to help me identify which part of the learning process I have not fully understood.
Collaboration orientation (Sidra and Mason, 2026)	C1	In academic tasks, I tend to use generative AI to improve my efficiency.
Collaboration orientation (Sidra and Mason, 2026)	C2	In academic tasks, I adjust the way I formulate my prompts so that generative AI produces outputs that better meet the task requirements.
Collaboration orientation (Sidra and Mason, 2026)	C3	In academic tasks, I tend to build upon and complement generative AI's output.
Collaboration orientation (Sidra and Mason, 2026)	C4	In academic tasks, I tend to determine what information generative AI needs to perform the task appropriately and provide it.
Collaboration orientation (Sidra and Mason, 2026)	C5	In academic tasks, I tend to try different ways of working with generative AI to achieve better outcomes.
Collaboration orientation (Sidra and Mason, 2026)	C6	In academic tasks, I tend to use generative AI to improve the quality of my decisions or work.
Critical openness (Sosu, 2013)	CO1	I usually try to think about the bigger picture during a discussion.
Critical openness (Sosu, 2013)	CO2	I often use new ideas to shape (modify) the way I do things.
Critical openness (Sosu, 2013)	CO3	I use more than one source to find out information for myself.
Critical openness (Sosu, 2013)	CO4	I am often on the lookout for new ideas.

Construct and source	Item	Final item wording
Critical openness (Sosu, 2013)	CO5	I sometimes find a good argument that challenges some of my firmly held beliefs.
Critical openness (Sosu, 2013)	CO6	It is important to understand other people's viewpoint on an issue.
Critical openness (Sosu, 2013)	CO7	It is important to justify the choices I make.
Reflective scepticism (Sosu, 2013)	RS1	I often re-evaluate my experiences so that I can learn from them.
Reflective scepticism (Sosu, 2013)	RS2	I usually check the credibility of the source of information before making judgements.
Reflective scepticism (Sosu, 2013)	RS3	I usually think about the wider implications of a decision before taking action.
Reflective scepticism (Sosu, 2013)	RS4	I often think about my actions to see whether I could improve them.

Note. The GenAI orientation items were rated from 1 (never) to 5 (almost always); CTD items were rated from 1 (not at all true of me) to 5 (very true of me).

Figure S1.

Prevalence of GenAI application use in the analytic sample

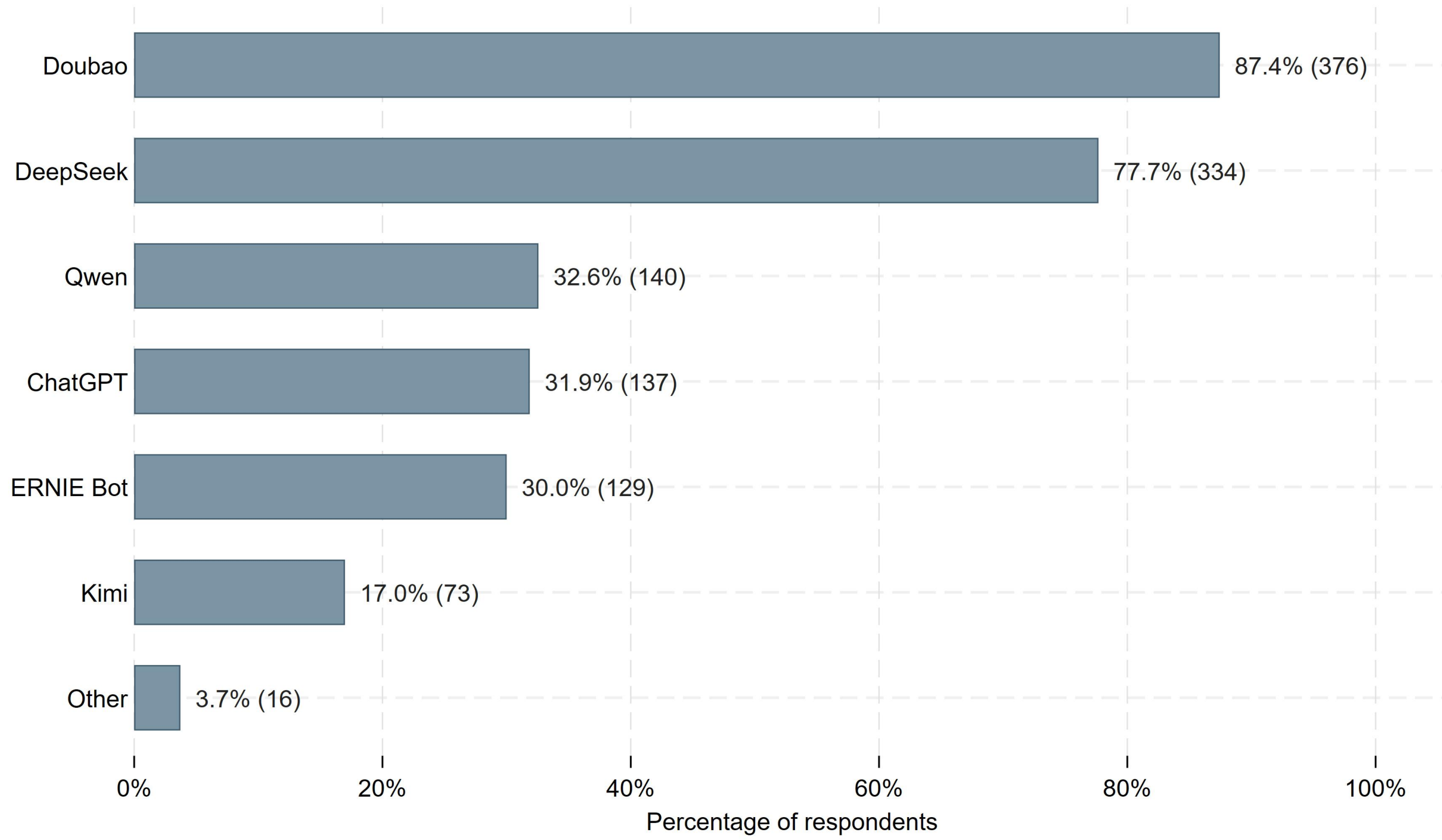

Note. Bars represent the percentage of respondents who selected each GenAI application; values in parentheses are frequencies. Respondents could select more than one application, so percentages do not sum to 100%. N = 430.

Figure S2.

Distribution of GenAI application use breadth by field of study

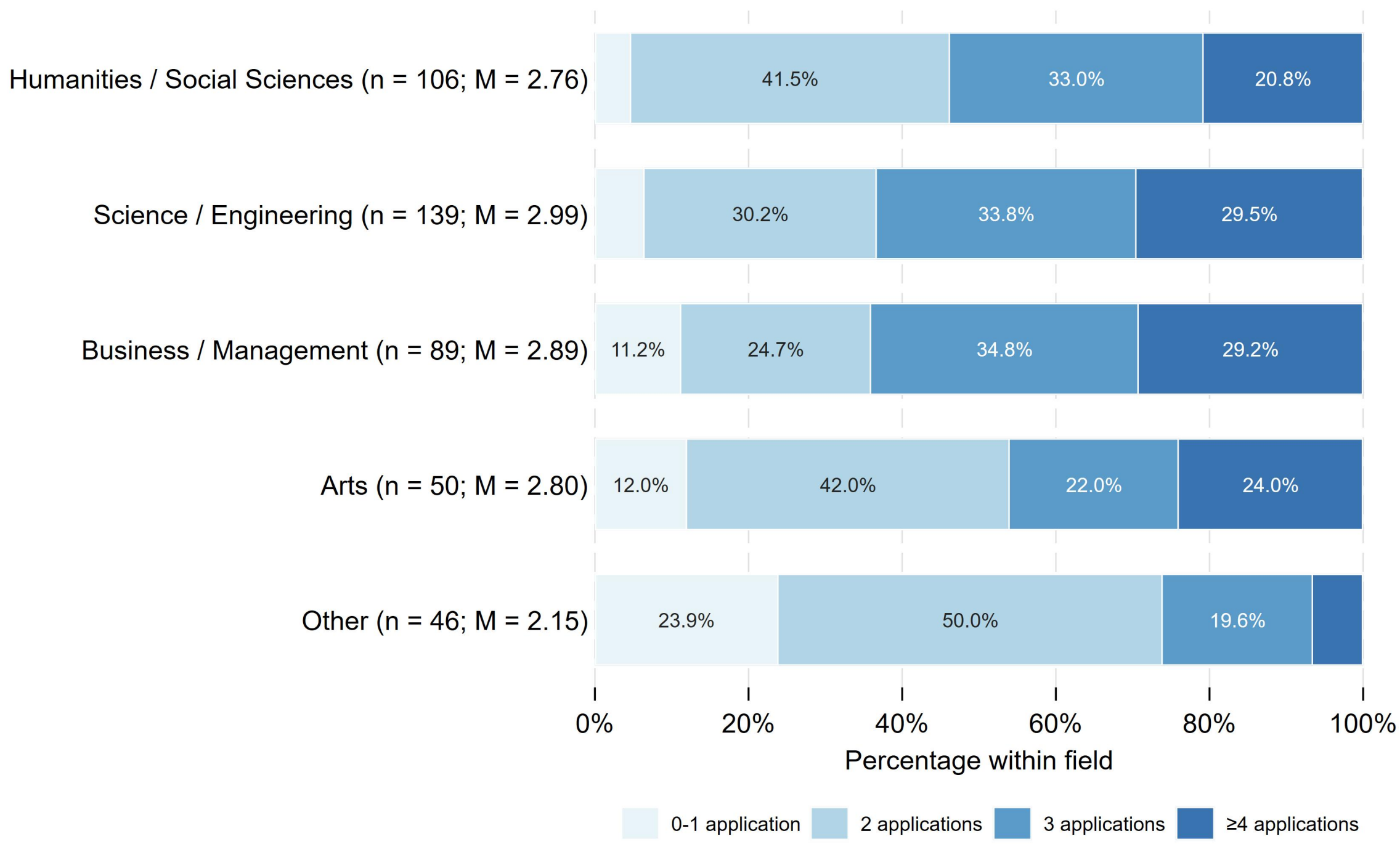

Note. GenAI application use breadth was defined as the number of GenAI applications selected by each respondent. Bars show percentages within fields and therefore sum to 100% for each field. The observed number of applications ranged from 0 to 6 and was grouped into 0–1, 2, 3, and 4 or more applications for graphical presentation. Parenthetical values give sample sizes for each field and unadjusted means. Fields follow the order used in Table 2. N = 430.

Table S2.

Full standardised factor loadings

Source model	Construct	Item	Standardised loading
Primary four-factor model	Tool orientation	T1	0.809
Primary four-factor model	Tool orientation	T2	0.827
Primary four-factor model	Tool orientation	T3	0.693
Primary four-factor model	Tool orientation	T4	0.743
Primary four-factor model	Guidance orientation	G1	0.822
Primary four-factor model	Guidance orientation	G2	0.770
Primary four-factor model	Guidance orientation	G3	0.701
Primary four-factor model	Guidance orientation	G4	0.719
Primary four-factor model	Collaboration orientation	C1	0.788
Primary four-factor model	Collaboration orientation	C2	0.738
Primary four-factor model	Collaboration orientation	C3	0.696
Primary four-factor model	Collaboration orientation	C4	0.751
Primary four-factor model	Collaboration orientation	C5	0.703
Primary four-factor model	Collaboration orientation	C6	0.729
Primary four-factor model	CTD	CO1	0.770
Primary four-factor model	CTD	CO2	0.771
Primary four-factor model	CTD	CO3	0.789
Primary four-factor model	CTD	CO4	0.792
Primary four-factor model	CTD	CO5	0.723
Primary four-factor model	CTD	CO6	0.724
Primary four-factor model	CTD	CO7	0.790
Primary four-factor model	CTD	RS1	0.814
Primary four-factor model	CTD	RS2	0.762
Primary four-factor model	CTD	RS3	0.789
Primary four-factor model	CTD	RS4	0.807
Original five-factor model	Critical openness	CO1	0.777
Original five-factor model	Critical openness	CO2	0.772
Original five-factor model	Critical openness	CO3	0.797
Original five-factor model	Critical openness	CO4	0.796
Original five-factor model	Critical openness	CO5	0.728
Original five-factor model	Critical openness	CO6	0.727
Original five-factor model	Critical openness	CO7	0.793
Original five-factor model	Reflective scepticism	RS1	0.822
Original five-factor model	Reflective scepticism	RS2	0.778
Original five-factor model	Reflective scepticism	RS3	0.797
Original five-factor model	Reflective scepticism	RS4	0.825

Table S3.

Full discriminant validity matrices

Panel A. Primary four-factor model

	Tool	Guidance	Collaboration	CTD
Tool	.770	.322	.303	.134
Guidance	.320	.755	.728	.432
Collaboration	.285	.731	.735	.579
CTD	−.060	.427	.573	.776

Panel B. Original five-factor model

	Tool	Guidance	Collaboration	CO	RS
Tool	.770	.322	.303	.129	.140
Guidance	.319	.755	.728	.423	.438
Collaboration	.285	.731	.735	.592	.544
CO	−.037	.417	.586	.771	.964
RS	−.091	.432	.540	.963	.806

Note. Diagonal entries are square roots of AVE. Entries below the diagonal are standardised latent correlations; entries above the diagonal are HTMT ratios.

Table S4.

Correlations involving the original CTD subscales

Variable	M	SD	1	2	3	4	5
1. Tool orientation	3.051	.870	—				
2. Guidance orientation	3.855	.715	.266***	—			
3. Collaboration orientation	3.870	.676	.254***	.622***	—		
4. Critical openness	3.877	.664	−.025	.369***	.527***	—	
5. Reflective scepticism	3.953	.696	−.072	.376***	.477***	.864***	—

Note. N = 430. Values below the diagonal are two-tailed Pearson correlations. *** $p < .001$.

Table S5.

Progression of the Tool orientation coefficient across regression specifications

Specification	B (HC3 SE)	95% CI for B	β (95% CI)	p	R^2
Tool alone	−.033 (.041)	[−.114, .048]	−.044 [−.151, .063]	.421	.002
Tool and covariates	−.081 (.036)	[−.152, −.011]	−.108 [−.202, −.014]	.024	.297
Tool, Guidance, and covariates	−.122 (.034)	[−.189, −.056]	−.163 [−.251, −.074]	< .001	.349
Tool, Collaboration, and covariates	−.132 (.033)	[−.197, −.067]	−.176 [−.262, −.090]	< .001	.381
Complete model	−.142 (.032)	[−.206, −.078]	−.189 [−.274, −.104]	< .001	.390

Note. N = 430 in every specification. Covariates comprised gender, educational level, field of study, duration of GenAI use, weekly use, and AI proficiency. The categorical covariates generated 14 indicator terms. All inferences used HC3 robust standard errors. The Tool-only model gives the same standardised coefficient as the Pearson correlation in Table 7. Its p value differs because Table S5 uses HC3 inference, while Table 7 uses the conventional Pearson correlation test.

Table S6.

Complete hierarchical regression coefficients predicting CTD

Predictor	Model 1	Model 2	Model 3
**Demographic controls**			
Female (ref: Male)	−.217*** (.063)	−.234*** (.063)	−.213*** (.059)
Vocational college (ref: Bachelor's)	−.505*** (.078)	−.375*** (.087)	−.127 (.086)
Postgraduate (ref: Bachelor's)	−.031 (.081)	−.086 (.079)	−.029 (.078)
Science/Engineering (ref: Humanities/Social Sciences)	−.062 (.077)	−.059 (.076)	−.068 (.071)
Business/Management (ref: Humanities/Social Sciences)	−.141 (.092)	−.131 (.086)	−.134 (.083)
Arts (ref: Humanities/Social Sciences)	−.068 (.094)	−.073 (.094)	−.126 (.085)
Other field (ref: Humanities/Social Sciences)	−.143 (.116)	−.098 (.120)	−.092 (.114)
**AI-use background**			
Never or <3 months (ref: >1 year)		−.223 (.148)	−.102 (.142)
3–6 months (ref: >1 year)		−.118 (.111)	−.059 (.108)
6–12 months (ref: >1 year)		−.010 (.063)	−.038 (.059)
No more than twice weekly (ref: Almost daily)		−.207* (.094)	−.121 (.100)
3–5 times weekly (ref: Almost daily)		−.070 (.064)	−.048 (.061)
Beginner (ref: Intermediate)		−.205* (.092)	−.167 (.087)
Advanced/expert (ref: Intermediate)		.028 (.067)	.015 (.062)
**GenAI usage orientations**			
Tool orientation			−.142*** (.032)
Guidance orientation			.115* (.048)
Collaboration orientation			.311*** (.080)
Constant	4.298*** (.062)	4.391*** (.076)	3.029*** (.363)

Note. Entries are unstandardised coefficients with HC3 robust standard errors in parentheses. * $p < .05$. *** $p < .001$. Model summaries appear in Table 8.

Table S7.

Fully adjusted CTD subscale models

Predictor	CO B (SE)	CO β [95% CI]	p	RS B (SE)	RS β [95% CI]	p
Tool orientation	−.125 (.033)	−.164 [−.250, −.078]	<.001	−.172 (.035)	−.215 [−.302, −.128]	<.001
Guidance orientation	.084 (.048)	.090 [−.011, .191]	.079	.169 (.056)	.174 [.061, .286]	.003
Collaboration orientation	.328 (.081)	.334 [.173, .495]	<.001	.281 (.085)	.273 [.110, .436]	.001
R^2 / adjusted R^2	.386 / .361			.344 / .317		
Model F	20.28		<.001	18.76		<.001

Note. N = 430. Both models include demographic characteristics, AI usage background, and the three orientations. B values use HC3 robust standard errors; standardised β confidence intervals come from models with standardised focal predictors and outcomes.
